# A High Resolution Color Image Restoration Algorithm for Thin TOMBO Imaging Systems

**DOI:** 10.3390/s90604649

**Published:** 2009-06-15

**Authors:** Amar A. El-Sallam, Farid Boussaid

**Affiliations:** School of Electrical, Electronic and Computer Engineering, The University of Western Australia, 35 Stirling Highway, Crawley, WA 6009, Australia; E-Mail: boussaid@ee.uwa.edu.au (F.B.); http://www.ee.uwa.edu.au

**Keywords:** image restoration, TOMBO, color imaging, CMOS imager, point operations, back-projection, cross-correlation, spectra

## Abstract

In this paper, we present a blind image restoration algorithm to reconstruct a high resolution (HR) color image from multiple, low resolution (LR), degraded and noisy images captured by thin (< 1*mm*) TOMBO imaging systems. The proposed algorithm is an extension of our grayscale algorithm reported in [1] to the case of color images. In this color extension, each Point Spread Function (PSF) of each captured image is assumed to be different from one color component to another and from one imaging unit to the other. For the task of image restoration, we use all spectral information in each captured image to restore each output pixel in the reconstructed HR image, i.e., we use the most efficient global category of point operations. First, the composite RGB color components of each captured image are extracted. A blind estimation technique is then applied to estimate the spectra of each color component and its associated blurring PSF. The estimation process is formed in a way that minimizes significantly the interchannel cross-correlations and additive noise. The estimated PSFs together with advanced interpolation techniques are then combined to compensate for blur and reconstruct a HR color image of the original scene. Finally, a histogram normalization process adjusts the balance between image color components, brightness and contrast. Simulated and experimental results reveal that the proposed algorithm is capable of restoring HR color images from degraded, LR and noisy observations even at low Signal-to-Noise Energy ratios (SNERs). The proposed algorithm uses FFT and only two fundamental image restoration constraints, making it suitable for silicon integration with the TOMBO imager.

## 1. Introduction

**Figure 1.**
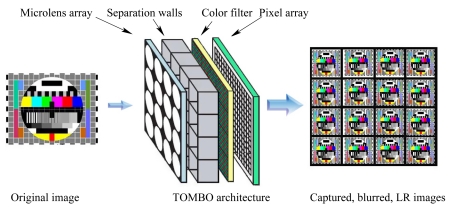
The architecture of a color TOMBO imaging system.

TOMBO (Thin Observation Module by Bound Optics) imaging systems are a new generation of paper-thin imaging products, which integrate micro-optics, photo-sensing elements and processing-circuitry all on one single silicon chip (Figure 1). These paper-thin imagers exhibit a thickness of less than a millimeter because they do not image the scene through a single imaging lens but through an array of microlenses [2]. The concept of a TOMBO imager was proposed and demonstrated by Tanida *et al.* [3–8]. The structure of a TOMBO imager is shown in Figure 1. It consists of an array of imaging units, each comprises a microlens that is associated with a subset of photo-sensitive pixel array. Individual imaging units are optically isolated by an opaque wall to prevent crosstalk (Figure 1). As a result, each individual imaging unit visualizes part of the scene. The output of the TOMBO imager is thus a mosaic of low resolution (LR) unit images. To reconstruct a high resolution (HR) image from the acquired set of LR images, Tanida *et al.* first proposed a Back-Projection (BP) method [6], which requires complete knowledge of the imaging system point spread function (PSF). The HR image of the scene is obtained by multiplying the captured LR images by the inverse (pseudo-inverse) of the known PSF. Tanida *et al.* proposed a second image reconstruction method (the “pixel rearrange method”) [7], which computes the cross-correlation peaks between captured unit images to arrange and align unit image pixels in the HR image of the scene. A de-shading pre-processing step is introduced to compensate for the shading introduced by the separation walls (Figure 1).

**Figure 2.**
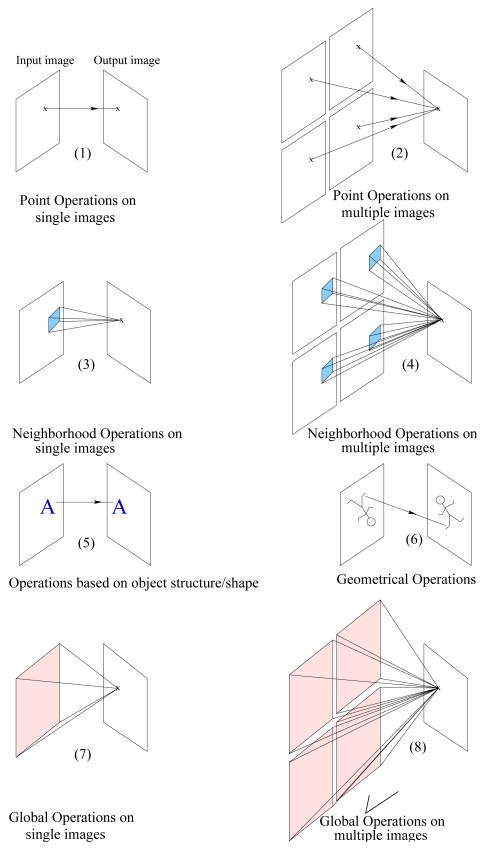
Point operations categories.

We have previously proposed a novel spectral-based image restoration algorithm that require neither prior information about the imaging system nor the original scene [1]. Furthermore, the proposed algorithm alleviates the need for de-shading and rearrangement processing. In this paper, we extend this algorithm to color images. We examine the difference in characteristics between grayscale and color images to develop a model for the color TOMBO imager. Previous work on color TOMBO imagers directly applied grayscale HR reconstruction algorithms to color images without considering the cross-correlation between color channels, and thus resulted in color artifacts [9–13]. In this paper, we exploit the global category of point operations for image restoration (see Figure 2) [14]. In this process, each pixel of the restored image is obtained by using information (pixels) from all captured LR images [15– 20].

The proposed spectral-based color image restoration method averages out all LR captured images, making the color channels globally independent of each other. Compared to previously reported color restoration techniques [9], this proposed algorithm uses FFT and only two fundamental image restoration constraints, which makes it suitable for silicon integration with a TOMBO imager. The paper is organized as follows: in section 2, we develop a model for a color TOMBO imager and derive the mathematical formulation of a novel blind color image restoration method. Section 3 details the color image restoration algorithm. Results are discussed in section 4. Finally, a conclusion is given in section 5.

## 2. Image Restoration Method for TOMBO Color Imaging Systems

In this section, we extend the grayscale image restoration algorithm reported in [1] to color TOMBO imagers. In the restoration process, we consider the global point operations based on multiple images. By using this category of point operations, we exploit all available information in the mosaic of simultaneously captured color images (see Figure 2). In addition, the global category is reported to have the ability to remove significant additive noise [15–20].

### 2.1. System Model

**Figure 3.**
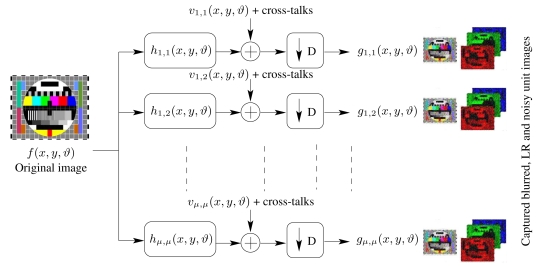
System model for the color TOMBO system.

Consider a TOMBO color system with (*μ* × *μ*) captured color images as shown in Figure 1. Each captured color image represents a blurred, LR and noisy observation of an original HR scene. The mathematical model (Figure 3) for the system can be described by

(1) $$\left[\begin{array}{c} g_{i,j}(x,y,R)\\ g_{i,j}(x,y,G)\\ g_{i,j}(x,y,B) \end{array}\right]=\left\{\left[\begin{array}{ccc} h_{i,j}(x,y,R) & h_{i,j}(x,y,GR) & h_{i,j}(x,y,BR)\\ h_{i,j}(x,y,GR) & h_{i,j}(x,y,G) & h_{i,j}(x,y,BG)\\ h_{i,j}(x,y,BR) & h_{i,j}(x,y,BG) & h_{i,j}(x,y,B) \end{array}\right]**\left[\begin{array}{c} f(x,y,R)\\ f(x,y,G)\\ f(x,y,B) \end{array}\right]+\left[\begin{array}{c} v_{i,j}(x,y,R)\\ v_{i,j}(x,y,G)\\ v_{i,j}(x,y,B) \end{array}\right]\right\}↓D$$

- *g_i,j_*(*x,y,ϑ*),*ϑ* ∈ {*R, G, B*} represents the blurred, LR and noisy color component for the *i^th^,j^th^* captured unit image with resolution (*M* × *N*) pixels per color
- *h_i,j_*(*x,y,ϑ*) is an (*l* × *l*) PSF that represents the overall channel blur affecting *g_i,j_*(*x,y,ϑ*) unit image for the color component *ϑ*, also called the intrachannel. We assume here that the blur is different for each color of each unit image
- *h_i,j_*(*x, y, GR*),*h_i,j_*(*x, y, BR*),*h_i,j_*(*x, y, BG*) are (*l* × *l*) PSFs representing the overall mutual relation between red-green, red-blue and green-blue respectively.
- “* *” represents the 2-D convolution operator w.r.t *x, y*
- *f*(*x, y, ϑ*) is the *ϑ* color component of the original scene with resolution (*M* × *N)* > (*M* × *N*) per color component
- *v_i,j_*(*x, y, ϑ*) is the additive 2-D, zero mean white Gaussian noise per color component that affect the unit image *g_i,j_*(*x,y,ϑ*)
- ↓ *D* is the down-sampling operator representing the LR process

Our main goal is to develop a restoration method that is able to reconstruct a HR, noiseless, color image of the original scene using only the (*μ* × *μ*) LR, blurred and noisy TOMBO color images *g_i,j_* (*x, y, ϑ*). The proposed method will have the following characteristics: (i) it does not require prior information about the imaging system nor the original scene, and thus performs blind image restoration, (ii) it can remove blur and additive noise from the HR color image, (iii) it exploits all available information contained in the captured LR images, and (iv) it requires minimal computational complexity.

### 2.2. Formulation of the Restoration Method

The general model of the color TOMBO imaging system is described by Equation (2). Let us consider all signal terms involved in the captured red color component of a unit image (*i, j*):

(2) $$\begin{array}{c} g_{i,j}(x,y,R)=[h_{i,j}(x,y,R)**f(x,y,R)\\ \;+h_{i,j}(x,y,GR)**f(x,y,G)\\ \;+h_{i,j}(x,y,BR)**f(x,y,B)\\ \;+v_{i,j}(x,y,R)]↓D,i,j=1,\ldots ,\mu \end{array}$$

By applying the two dimensional *ᵶ*-transform to the model in Equation (2), we have

(3) $$\begin{array}{c} G_{i,j}(z_{1},z_{2},R)=\frac{1}{D^{2}}\sum_{k=0}^{D-1}\sum_{l=0}^{D-1}H_{i,j}\left(z_{1}^{\frac{1}{D}}e^{\frac{-j2\pi k}{D}},z_{2}^{\frac{1}{D}}e^{\frac{-j2\pi l}{D}},R\right)F\left(z_{1}^{\frac{1}{D}}e^{\frac{-j2\pi k}{D}},z_{2}^{\frac{1}{D}}e^{\frac{-j2\pi l}{D}},R\right)\\ \;+\frac{1}{D^{2}}\sum_{k=0}^{D-1}\sum_{l=0}^{D-1}H_{i,j}\left(z_{1}^{\frac{1}{D}}e^{\frac{-j2\pi k}{D}},z_{2}^{\frac{1}{D}}e^{\frac{-j2\pi l}{D}},GR\right)F\left(z_{1}^{\frac{1}{D}}e^{\frac{-j2\pi k}{D}},z_{2}^{\frac{1}{D}}e^{\frac{-j2\pi l}{D}},GR\right)\\ \;+\frac{1}{D^{2}}\sum_{k=0}^{D-1}\sum_{l=0}^{D-1}H_{i,j}\left(z_{1}^{\frac{1}{D}}e^{\frac{-j2\pi k}{D}},z_{2}^{\frac{1}{D}}e^{\frac{-j2\pi l}{D}},BR\right)F\left(z_{1}^{\frac{1}{D}}e^{\frac{-j2\pi k}{D}},z_{2}^{\frac{1}{D}}e^{\frac{-j2\pi l}{D}},BR\right)\\ \;+\frac{1}{D^{2}}\sum_{k=0}^{D-1}\sum_{l=0}^{D-1}V_{i,j}\left(z_{1}^{\frac{1}{D}}e^{\frac{-j2\pi k}{D}},z_{2}^{\frac{1}{D}}e^{\frac{-j2\pi l}{D}},R\right), \end{array}$$

where $z_{1}=e^{j2\pi f_{1}}$, $z_{2}=e^{j2\pi f_{2}}$*andG_i,j_*(*z*_1_, *z*_2_, *R*), is the *ᵶ*-transform of *g_i,j_*(*x, y, R*).

The system model in Equation (3) is partitioned into the following terms:

(4) $$\begin{array}{c} G_{i,j}(z_{1},z_{2},R)=T_{i,j}^{a}\left(z_{1}^{\frac{1}{D}},z_{2}^{\frac{1}{D}},R\right)+T_{i,j}^{b}\left(z_{1}^{\frac{1}{D}},z_{2}^{\frac{1}{D}},R\right)+T_{i,j}^{c}\left(z_{1}^{\frac{1}{D}},z_{2}^{\frac{1}{D}},R\right)\\ +T_{i,j}^{d}\left(z_{1}^{\frac{1}{D}},z_{2}^{\frac{1}{D}},GR\right)+T_{i,j}^{e}\left(z_{1}^{\frac{1}{D}},z_{2}^{\frac{1}{D}},BR\right) \end{array}$$

where,

- $T_{i,j}^{a}\left(z_{1}^{\frac{1}{D}},z_{2}^{\frac{1}{D}},R\right)=H_{i,j}\left(z_{1}^{\frac{1}{D}},z_{2}^{\frac{1}{D}},R\right)F\left(z_{1}^{\frac{1}{D}},z_{2}^{\frac{1}{D}},R\right)+V_{i,j}\left(z_{1}^{\frac{1}{D}},z_{2}^{\frac{1}{D}},R\right)$ represents the image of interest plus the noise term (defined in-frequency band useful terms),
- $T_{i,j}^{b}\left(z_{1}^{\frac{1}{D}},z_{2}^{\frac{1}{D}},R\right)=\sum_{k=1}^{D-1}\sum_{l=1}^{D-1}H_{i,j}\left(z_{1}^{\frac{1}{D}}e^{\frac{-j2\pi k}{D}},z_{2}^{\frac{1}{D}}e^{\frac{-j2\pi l}{D}},R\right)F\left(z_{1}^{\frac{1}{D}}e^{\frac{-j2\pi k}{D}},z_{2}^{\frac{1}{D}}e^{\frac{-j2\pi l}{D}},R\right)$, are the aliasing out of frequency band image terms,
- $T_{i,j}^{c}\left(z_{1}^{\frac{1}{D}},z_{2}^{\frac{1}{D}},R\right)=\sum_{k=1}^{D-1}\sum_{l=1}^{D-1}V_{i,j}\left(z_{1}^{\frac{1}{D}}e^{\frac{-j2\pi k}{D}},z_{2}^{\frac{1}{D}}e^{\frac{-j2\pi l}{D}},R\right)$, are the aliasing out-of-frequency band noise terms.
- $T_{i,j}^{d}\left(z_{1}^{\frac{1}{D}},z_{2}^{\frac{1}{D}},GR\right)=\sum_{k=1}^{D-1}\sum_{l=1}^{D-1}H_{i,j}\left(z_{1}^{\frac{1}{D}}e^{\frac{-j2\pi k}{D}},z_{2}^{\frac{1}{D}}e^{\frac{-j2\pi l}{D}},GR\right)F\left(z_{1}^{\frac{1}{D}}e^{\frac{-j2\pi k}{D}},z_{2}^{\frac{1}{D}}e^{\frac{-j2\pi l}{D}},G\right)$, are the GR overall cross-talk terms.
- $T_{i,j}^{e}\left(z_{1}^{\frac{1}{D}},z_{2}^{\frac{1}{D}},BR\right)=\sum_{k=1}^{D-1}\sum_{l=1}^{D-1}H_{i,j}\left(z_{1}^{\frac{1}{D}}e^{\frac{-j2\pi k}{D}},z_{2}^{\frac{1}{D}}e^{\frac{-j2\pi l}{D}},BR\right)F\left(z_{1}^{\frac{1}{D}}e^{\frac{-j2\pi k}{D}},z_{2}^{\frac{1}{D}}e^{\frac{-j2\pi l}{D}},B\right)$, are the BR overall cross-talk terms.

In the above terms, the constant $\frac{1}{D^{2}}$ is not shown for simplification.

By multiplying both sides of Equation (4) with the complex conjugate of the red (*i,j*) blurring PSF, $H_{i,j}\left(z_{1}^{\frac{1}{D}},z_{2}^{\frac{1}{D}},R\right)$, i.e., $H_{i,j}^{*}\left(z_{1}^{\frac{1}{D}},z_{2}^{\frac{1}{D}},R\right)$ and similarly by $F*\left(z_{1}^{\frac{1}{D}},z_{2}^{\frac{1}{D}},R\right)$ and applying the ensemble average operator, E{}, we have,

(5) $$\begin{array}{c} E\left\{G_{i,j}H_{i,j}^{*}\left(z_{1}^{\frac{1}{D}},z_{2}^{\frac{1}{D}},R\right)\right\}=E\left\{T_{i,j}^{a}H_{i,j}^{*}\left(z_{1}^{\frac{1}{D}},z_{2}^{\frac{1}{D}},R\right)\right\}+E\left\{T_{i,j}^{b}H_{i,j}^{*}\left(z_{1}^{\frac{1}{D}},z_{2}^{\frac{1}{D}},R\right)\right\}+E\left\{T_{i,j}^{c}H_{i,j}^{*}\left(z_{1}^{\frac{1}{D}},z_{2}^{\frac{1}{D}},R\right)\right\}\\ +E\left\{T_{i,j}^{d}H_{i,j}^{*}\left(z_{1}^{\frac{1}{D}},z_{2}^{\frac{1}{D}},GR\right)\right\}+E\left\{T_{i,j}^{e}H_{i,j}^{*}\left(z_{1}^{\frac{1}{D}},z_{2}^{\frac{1}{D}},BR\right)\right\}\\ E\left\{G_{i,j}F*\left(z_{1}^{\frac{1}{D}},z_{2}^{\frac{1}{D}},R\right)\right\}=E\left\{T_{i,j}^{a}F*\left(z_{1}^{\frac{1}{D}},z_{2}^{\frac{1}{D}},R\right)\right\}+E\left\{T_{i,j}^{b}F*\left(z_{1}^{\frac{1}{D}},z_{2}^{\frac{1}{D}},R\right)\right\}+E\left\{T_{i,j}^{c}F*\left(z_{1}^{\frac{1}{D}},z_{2}^{\frac{1}{D}},R\right)\right\}\\ +E\left\{T_{i,j}^{d}F*\left(z_{1}^{\frac{1}{D}},z_{2}^{\frac{1}{D}},GR\right)\right\}+E\left\{T_{i,j}^{e}F*\left(z_{1}^{\frac{1}{D}},z_{2}^{\frac{1}{D}},BR\right)\right\} \end{array}$$

which leads to:

(6) $$\begin{array}{c} C_{G_{i,j}H_{i,j}^{*}}\left(z_{1}^{\frac{1}{D}},z_{2}^{\frac{1}{D}},R\right)=F\left(z_{1}^{\frac{1}{D}},z_{2}^{\frac{1}{D}},R\right)C_{H_{i,j}H_{i,j}^{*}}\left(z_{1}^{\frac{1}{D}},z_{2}^{\frac{1}{D}},R\right)+C_{V_{i,j}H_{i,j}^{*}}\left(z_{1}^{\frac{1}{D}},z_{2}^{\frac{1}{D}},R\right)\\ +C_{T_{i,j}^{b}H_{i,j}^{*}}\left(z_{1}^{\frac{1}{D}},z_{2}^{\frac{1}{D}},R\right)+C_{T_{i,j}^{c}H_{i,j}^{*}}\left(z_{1}^{\frac{1}{D}},z_{2}^{\frac{1}{D}},R\right)\\ +C_{T_{i,j}^{d}H_{i,j}^{*}}\left(z_{1}^{\frac{1}{D}},z_{2}^{\frac{1}{D}},GR\right)+C_{T_{i,j}^{e}H_{i,j}^{*}}\left(z_{1}^{\frac{1}{D}},z_{2}^{\frac{1}{D}},BR\right)\\ C_{G_{i,j}F*}\left(z_{1}^{\frac{1}{D}},z_{2}^{\frac{1}{D}},R\right)=H_{i,j}^{*}\left(z_{1}^{\frac{1}{D}},z_{2}^{\frac{1}{D}},R\right)C_{FF*}\left(z_{1}^{\frac{1}{D}},z_{2}^{\frac{1}{D}},R\right)+C_{V_{i,j}F*}\left(z_{1}^{\frac{1}{D}},z_{2}^{\frac{1}{D}},R\right)\\ +C_{T_{i,j}^{b}F*}\left(z_{1}^{\frac{1}{D}},z_{2}^{\frac{1}{D}},R\right)+C_{T_{i,j}^{c}F*}\left(z_{1}^{\frac{1}{D}},z_{2}^{\frac{1}{D}},R\right)\\ +C_{T_{i,j}^{d}F*}\left(z_{1}^{\frac{1}{D}},z_{2}^{\frac{1}{D}},GR\right)+C_{T_{i,j}^{e}F*}\left(z_{1}^{\frac{1}{D}},z_{2}^{\frac{1}{D}},BR\right) \end{array}$$

where cross-spectra C_XY*_ (*z*_1_, *z*_2_, *R*) = E {*X*(*z*_1_, *z*_2_, *R*)Y* (*z*_1_, *z*_2_, *R*)} over *z*_1_, *z*_2_.

Since the frequency bandwidth of the blurring PSF function *H_i,j_*(*z*_1_, *z*_2_, *R*) is limited [15, 19, 21], the terms $T_{i,j}^{b}\left(z_{1}^{\frac{1}{D}},z_{2}^{\frac{1}{D}},R\right)$ and $T_{i,j}^{c}\left(z_{1}^{\frac{1}{D}},z_{2}^{\frac{1}{D}},R\right)$ are not in the same frequency band where $H_{i,j}^{*}\left(z_{1}^{\frac{1}{D}},z_{2}^{\frac{1}{D}},R\right)$ is. Therefore, the last two cross-spectral terms of Equation (6) can be discarded, leading to:

(7) $$\begin{array}{c} C_{G_{i,j}H_{i,j}^{*}}\left(z_{1}^{\frac{1}{D}},z_{2}^{\frac{1}{D}},R\right)=F\left(z_{1}^{\frac{1}{D}},z_{2}^{\frac{1}{D}},R\right)C_{H_{i,j}H_{i,j}^{*}}\left(z_{1}^{\frac{1}{D}},z_{2}^{\frac{1}{D}},R\right)+C_{V_{i,j}H_{i,j}^{*}}\left(z_{1}^{\frac{1}{D}},z_{2}^{\frac{1}{D}},R\right)\\ +C_{T_{i,j}^{d}H_{i,j}^{*}}\left(z_{1}^{\frac{1}{D}},z_{2}^{\frac{1}{D}},GR\right)+C_{T_{i,j}^{e}H_{i,j}^{*}}\left(z_{1}^{\frac{1}{D}},z_{2}^{\frac{1}{D}},BR\right)\\ C_{G_{i,j}F*}\left(z_{1}^{\frac{1}{D}},z_{2}^{\frac{1}{D}},R\right)=H_{i,j}^{*}\left(z_{1}^{\frac{1}{D}},z_{2}^{\frac{1}{D}},R\right)C_{FF*}\left(z_{1}^{\frac{1}{D}},z_{2}^{\frac{1}{D}},R\right)+C_{V_{i,j}F*}\left(z_{1}^{\frac{1}{D}},z_{2}^{\frac{1}{D}},R\right)\\ +C_{T_{i,j}^{d}F*}\left(z_{1}^{\frac{1}{D}},z_{2}^{\frac{1}{D}},GR\right)+C_{T_{i,j}^{e}F*}\left(z_{1}^{\frac{1}{D}},z_{2}^{\frac{1}{D}},BR\right) \end{array}$$

To minimize the effects due to interchannel cross-correlation terms (cross-talks) $C_{T_{i,j}^{d}H_{i,j}^{*}}\left(z_{1}^{\frac{1}{D}},z_{2}^{\frac{1}{D}},R\right)$ and $C_{T_{i,j}^{e}H_{i,j}^{*}}\left(z_{1}^{\frac{1}{D}},z_{2}^{\frac{1}{D}},R\right)$ in Equation (7), a typical approach would be to apply a decorrelation stretch technique to the captured LR images [14, 22]. However, such technique uses principal component analysis, which is computationally expensive and thus not suited for silicon integration with a TOMBO imager. An alternative solution is to minimize the cross-correlation terms by using ergodicity, one of the wide-sense stationary properties of signals [23]. Let us consider the cross-correlations between the color channels, which depend on the reflection characteristics of the imaged objects. If the object exhibits strong reflection at the region of correlated wavelengths, then the cross-correlation between the channels could be strong. However, since the characteristics of one object is almost independent of those of other objects, the averaged interchannel cross-correlations over the entire image can be very weak. As a result, the RGB components of a color image can be regarded as being locally correlated but globally independent of each other [23]. In this paper, we exploit this property by using a global category of point operations for image restoration. In this process, each pixel of the restored image is obtained by using information (pixels) from all captured LR images (Figure 2). This is carried out by computing the cross-spectra between the different color channels. Since the cross-spectra are nothing but the Fourier transform of the cross-correlations, our algorithm essentially averages out the interchannel cross-correlations over the entire image. As a result, the averaged interchannel cross-correlations become very weak [23]. Our spectral-based restoration method has the effect of further minimizing the interchannel cross-correlations because it averages out over all captured LR images. As a result, the cross-spectral terms of Equation 7, $C_{T_{i,j}^{d}H_{i,j}^{*}}\left(z_{1}^{\frac{1}{D}},z_{2}^{\frac{1}{D}},GR\right)$ the $C_{T_{i,j}^{e}H_{i,j*}^{*}}\left(z_{1}^{\frac{1}{D}},z_{2}^{\frac{1}{D}},BR\right)$, $C_{T_{i,j}^{d}F*}\left(z_{1}^{\frac{1}{D}},z_{2}^{\frac{1}{D}},GR\right)$, $C_{T_{i,j}^{e}F*}\left(z_{1}^{\frac{1}{D}},z_{2}^{\frac{1}{D}},BR\right)$ can be neglected, leading to:

(8) $$\begin{array}{c} C_{G_{i,j}H_{i,j}^{*}}\left(z_{1}^{\frac{1}{D}},z_{2}^{\frac{1}{D}},R\right)=F\left(z_{1}^{\frac{1}{D}},z_{2}^{\frac{1}{D}},R\right)C_{H_{i,j}H_{i,j}^{*}}\left(z_{1}^{\frac{1}{D}},z_{2}^{\frac{1}{D}},R\right)+C_{V_{i,j}H_{i,j}^{*}}\left(z_{1}^{\frac{1}{D}},z_{2}^{\frac{1}{D}},R\right)\\ C_{G_{i,j}F*}\left(z_{1}^{\frac{1}{D}},z_{2}^{\frac{1}{D}},R\right)=H_{i,j}\left(z_{1}^{\frac{1}{D}},z_{2}^{\frac{1}{D}},R\right)C_{FF*}\left(z_{1}^{\frac{1}{D}},z_{2}^{\frac{1}{D}},R\right)+C_{V_{i,j}F*}\left(z_{1}^{\frac{1}{D}},z_{2}^{\frac{1}{D}},R\right) \end{array}$$

where $C_{V_{i,j}H_{i,j}^{*}}\left(z_{1}^{\frac{1}{D}},z_{2}^{\frac{1}{D}},R\right)$ and $C_{V_{i,j}F*}\left(z_{1}^{\frac{1}{D}},z_{2}^{\frac{1}{D}},R\right)$ are the residual errors representing the in-band cross spectral terms between original images, in the PSFs and in the independent additive noise respectively. These errors can be assumed 2-D, independent and identically distributed (i.i.d.) signals under some regularity conditions [24]. By extending the previous analysis to the two other color components G and B, (8) can be generalized:

(9) $$\begin{array}{c} \left[\begin{array}{c} C_{G_{i,j}H_{i,j}^{*}}\left(z_{1}^{\frac{1}{D}},z_{2}^{\frac{1}{D}},R\right)\\ C_{G_{i,j}H_{i,j}^{*}}\left(z_{1}^{\frac{1}{D}},z_{2}^{\frac{1}{D}},G\right)\\ C_{G_{i,j}H_{i,j}^{*}}\left(z_{1}^{\frac{1}{D}},z_{2}^{\frac{1}{D}},B\right) \end{array}\right]\approx \left[\begin{array}{ccc} C_{H_{i,j}H_{i,j}^{*}}\left(z_{1}^{\frac{1}{D}},z_{2}^{\frac{1}{D}},R\right) & 0 & 0\\ 0 & C_{H_{i,j}H_{i,j}^{*}}\left(z_{1}^{\frac{1}{D}},z_{2}^{\frac{1}{D}},G\right) & 0\\ 0 & 0 & C_{H_{i,j}H_{i,j}^{*}}\left(z_{1}^{\frac{1}{D}},z_{2}^{\frac{1}{D}},B\right) \end{array}\right]\;[\begin{array}{c} F\left(z_{1}^{\frac{1}{D}},z_{2}^{\frac{1}{D}},R\right)\\ F\left(z_{1}^{\frac{1}{D}},z_{2}^{\frac{1}{D}},G\right)\\ F\left(z_{1}^{\frac{1}{D}},z_{2}^{\frac{1}{D}},B\right) \end{array}]\\ +\left[\begin{array}{c} C_{V_{i,j}H_{i,j}^{*}}\left(z_{1}^{\frac{1}{D}},z_{2}^{\frac{1}{D}},R\right)\\ C_{V_{i,j}H_{i,j}^{*}}\left(z_{1}^{\frac{1}{D}},z_{2}^{\frac{1}{D}},G\right)\\ C_{V_{i,j}H_{i,j}^{*}}\left(z_{1}^{\frac{1}{D}},z_{2}^{\frac{1}{D}},B\right) \end{array}\right] \end{array}$$

and similarly

(10) $$\begin{array}{c} \left[\begin{array}{c} C_{G_{i,j}F*}\left(z_{1}^{\frac{1}{D}},z_{2}^{\frac{1}{D}},R\right)\\ C_{G_{i,j}F*}\left(z_{1}^{\frac{1}{D}},z_{2}^{\frac{1}{D}},G\right)\\ C_{G_{i,j}F*}\left(z_{1}^{\frac{1}{D}},z_{2}^{\frac{1}{D}},B\right) \end{array}\right]\approx \left[\begin{array}{ccc} C_{FF*}\left(z_{1}^{\frac{1}{D}},z_{2}^{\frac{1}{D}},R\right) & 0 & 0\\ 0 & C_{FF*}\left(z_{1}^{\frac{1}{D}},z_{2}^{\frac{1}{D}},G\right) & 0\\ 0 & 0 & C_{FF*}\left(z_{1}^{\frac{1}{D}},z_{2}^{\frac{1}{D}},B\right) \end{array}\right]\;[\begin{array}{c} H_{i,j}^{*}\left(z_{1}^{\frac{1}{D}},z_{2}^{\frac{1}{D}},R\right)\\ H_{i,j}^{*}\left(z_{1}^{\frac{1}{D}},z_{2}^{\frac{1}{D}},G\right)\\ H_{i,j}^{*}\left(z_{1}^{\frac{1}{D}},z_{2}^{\frac{1}{D}},B\right) \end{array}]\\ +\left[\begin{array}{c} C_{V_{i,j}F*}\left(z_{1}^{\frac{1}{D}},z_{2}^{\frac{1}{D}},R\right)\\ C_{V_{i,j}F*}\left(z_{1}^{\frac{1}{D}},z_{2}^{\frac{1}{D}},G\right)\\ C_{V_{i,j}F*}\left(z_{1}^{\frac{1}{D}},z_{2}^{\frac{1}{D}},B\right) \end{array}\right] \end{array}$$

### 2.3. Restoration Process

From Equations (8), (9), and (10), if some prior information or constraints about either the original or the blur is given, it is then possible to restore the original image. This can be done independently for each color component or jointly for all color components using Equations (9), and (10). For example, if the PSF associated with each captured *g_i,j_*(*x,y, R*) unit image, $H_{i,j}\left(z_{1}^{\frac{1}{D}},z_{2}^{\frac{1}{D}},R\right)$, is pre-determined or can be estimated, then it becomes possible to restore the original image using,

(11) $$F\left(z_{1}^{\frac{1}{D}},z_{2}^{\frac{1}{D}},R\right)=\frac{C_{G_{i,j}H_{i,j}^{*}}\left(z_{1},z_{2},R\right)-C_{V_{i,j}H_{i,j}^{*}}\left(z_{1}^{\frac{1}{D}},z_{2}^{\frac{1}{D}},R\right)}{C_{H_{i,j}H_{i,j}^{*}}\left(z_{1}^{\frac{1}{D}},z_{2}^{\frac{1}{D}},R\right)}$$

The above equation is valid under some constraints [24, 25]. One constraint is that the number of zeros in *H_i,j_*(*z*_1_, *z*_2_, *R*) is much less than the size of the image. Because our method sums all PSFs spectra when implementing Equation (11), this will minimize the probability of having a division by zero. In general, if zeros exist but only in small numbers compared to the image size, a tolerance value can be added to the denominator to avoid division by zero. Other problems associated with image and PSF restoration methods are (i) the length *£* and the PSF are unknown and most critically (ii) the impact of residual error terms, which can significantly deteriorate the restoration process [26, 27]. However, since a TOMBO imaging system can capture multiple observations of the same scene, it is possible to reduce the effects of the error terms significantly by using averaged cross-spectral techniques [25]. The use of global point operations will also minimize the additive noise [15–20]. To clarify this point, consider an imaging system with (*μ × μ*) captured images, the averaged spectral and cross-spectral techniques can be then applied to provide results similar to Equation (8) using spectral estimates instead of the true estimates. In mathematical form, $F\left(z_{1}^{\frac{1}{D}},z_{2}^{\frac{1}{D}},R\right)$ and similarly $H_{i,j}\left(z_{1}^{\frac{1}{D}},z_{2}^{\frac{1}{D}},R\right)$ can be estimated using the averaged cross-spectra defined by

(12) $$\sum_{i=1}^{\mu }\sum_{j=1}^{\mu }{\hat{C}}_{G_{i,j}H_{i,j}^{*}}(z_{1},z_{2},R)=F\left(z_{1}^{\frac{1}{D}},z_{2}^{\frac{1}{D}},R\right)\sum_{i=1}^{\mu }\sum_{j=1}^{\mu }{\hat{C}}_{H_{i.j}H_{i,j}^{*}}\left(z_{1}^{\frac{1}{D}},z_{2}^{\frac{1}{D}},R\right)+\sum_{i=1}^{\mu }\sum_{j=1}^{\mu }{\hat{C}}_{V_{i,j}H_{i,j}^{*}}\left(z_{1}^{\frac{1}{D}},z_{2}^{\frac{1}{D}},R\right)$$

where Ĉ_XY*_(*z*_1_,*z*_2_,*R*) *= X*(*z*_1_,*z_2_, R*) Y*(*z*_1_, *z_2_, R*) is an estimate of the cross-spectra between *X*(*z*_1_, *z*_2_, *R*) and *Y*(*z*_1_,*z_2_, R*). For sufficient number of *μ* images, the last summation (error term of Equation (12)), is nothing but the mean value of an i.i.d. signal which has a zero mean [24, 25]. In this situation and after employing interpolation techniques, we have:

(13) $$\hat{F}(z_{1},z_{2},R)\approx \frac{\sum_{i=1}^{\mu }\sum_{j=1}^{\mu }{\hat{C}}_{G_{i,j}H_{i,j}^{*}}(z_{1},z_{2},R)}{\sum_{i=1}^{\mu }\sum_{j=1}^{\mu }{\hat{C}}_{H_{i,j}H_{i,j}^{*}}(z_{1},z_{2},R)}$$

Under some constraints [17, 19, 20, 22, 25-28], the above estimates can be used to restore the original image.

## 3. Color Image Restoration Algorithm

In this section, we employ the work formulated above to develop a color image restoration algorithm. Using Equation (13), it is possible to restore the original image using iterative techniques. During the restoration process, the algorithm will only impose two fundamental image restoration constraints (positivity and support region):

- For restoring the image
  (14) $$\hat{f}(x,y,R)=\{\begin{array}{cc} \left|\hat{f}(x,y,R)\right|, & <x,y>\in \left[LM,LN\right]\\ F_{M}, & \left|\hat{f}(x,y,R)\right|\geq F_{\infty },<x,y>\in \left[LM,LN\right]\\ 0 & \text{otherwise} \end{array}$$
  where, *F_M_* is the mean value of a mesh of pixels in the region < x, y > ∈ [LM, LN] surrounding symmetrically the pixel index *(x, y), F*∞ is a threshold of a large value pre-defined for the case when the summation of the PSF spectra in Equation 13 is close to zero.
- For estimating the PSFs
  (15) $${\hat{h}}_{i,j}(x,y,R)=\{\underset{0}{\left|{\hat{h}}_{i,j}(x,y,R)\right|},\underset{\text{otherwise}}{<x,y,\in \left[\ell ,\ell \right]}$$

where, *L* ≥ *D* is the interpolation or up-sampling factor needed to restore the HR image, (*LM* × *LN*) is the size of the restored image which can be greater or equal to the size of the original image (*M × N*), and (ℓ × ℓ) is the size of the estimated PSF.

Practical considerations for the iterative algorithm implementation can be summarized as follows:

- Pixel amplitudes that reach values greater than 255 are scaled using the following histogram normalization,
  (16) $${\hat{f}}_{\text{scaled}}(x,y,R)=a\times \frac{\left(\hat{f}(x,y,R)-f_{\text{min}}R\right)}{\left(f_{\text{max},R}-f_{\text{min},R}\right)}+b$$
  where *a* and *b* are usually 255 and 0 respectively (but other values can be also used to adjust contrast and brightness), *f_max, R_* and *f_min, R_* are the maximum and minimum pixel values of the color component *R.* For improved performance, *f_mm_,_R_* and *f_max_,_R_* are usually chosen as the 5% and 95% levels in the histogram distribution respectively (confidence interval).
- The mean value of the input image(s) and the output image is to be maintained (note that there are twice as many green pixels as red/blue pixel for the Bayer filter)
- To resolve the problem of having zeros or nulls in the spectra, the following equation for the interpolated *f*(*x,y, R*) is used:
  (17) $$\tilde{F}(z_{1},z_{2},R)\approx \frac{\sum_{i=1}^{\mu }\sum_{j=1}^{\mu }{\hat{C}}_{G_{i,j}H_{i,j}^{*}}(z_{1},z_{2},R)-\alpha_{1}}{\sum_{i=1}^{\mu }\sum_{j=1}^{\mu }{\hat{C}}_{H_{i,j}H_{i,j}^{*}}(z_{1},z_{2},R)-\alpha_{2}}$$
  followed by:
  (18) $${\hat{F}}^{\left(n+1\right)}(z_{1},z_{2},R)=\beta {\hat{F}}^{\left(n\right)}\left(z_{1},z_{2},R\right)+\left(1-\beta \right)\hat{F}(z_{1},z_{2},R)$$
  where *α*_1_,*α_2_* are two positive numbers representing the extent of additive noise in the residual terms, *β* is a recursive stability factor controls the amount of information needed from posterior image estimates (0 *< β <* 1 ), *n* + 1 is the current iteration number. Typical values of *β* range between 0.5 to 0.9. Like in adaptive systems, the value of *β* can be adjusted to avoid such that impulsive-like outputs.
- For initialization, one of the images is used as an initial estimate of the HR image. The up-sampling and interpolation process is done by zero-padding in the spatial domain between the image samples. Afterwards the FFT is applied. In the Fourier domain, a single spectrum is then taken out of the repetitive spectra using a low pass filter with cut-off frequency $\left(\frac{\pi }{L}\right)$ and zeroing the rest of the spectrum. Finally, inverse fast Fourier transform (IFFT) is applied to inverse back to the image domain. It is essential that the zero-padding be done such that the zero frequency components remain the same. In addition, zero-padding should be applied to both positive and negative frequencies. Unlike existing techniques that use lower order functions for interpolation (cubic interpolation used in [2]), our method uses the more efficient sinc function.
- We use the 2-D fast fourier transform (FFT) to estimate spectra and cross spectra needed for the algorithm

The proposed HR color image restoration algorithm is detailed in Table 1.

### 3.1. Convergence Analysis

From Equation (18) and after considering the situation where *n* = 0,1,2,… ,*n_o_* (note that *F̃* also changes at each iteration), after some mathematical manipulation it can be shown that

(19) $${\hat{F}}^{\left(n_{0}+1\right)}(z_{1},z_{2},R)=\beta^{\left(n_{0}+1\right)}{\hat{F}}^{\left(0\right)}(z_{1},z_{2},R)+\sum_{j=0}^{n_{0}}\beta^{j}(1-\beta ){\tilde{F}}^{\left(n_{0}-j\right)}(z_{1},z_{2},R)$$

For a value of 0 *< β <* 1 and when the number of iteration *n_o_* → ∞ or large enough, the above equation can be approximated to:

(20) $${\hat{F}}^{\left(n_{0}+1\right)}(z_{1},z_{2},R)=\sum_{j_{0}}^{n_{0}}\beta^{j}(1-\beta ){\tilde{F}}^{\left(n_{0}-j\right)}(z_{1},z_{2},R)$$

Based on spectral estimation principals [24, 25], and by using the image estimator provided in Equation (17), it can be proven that:

(21) $$E\left\{\hat{F}(z_{1},z_{2},R)\right\}=F(z_{1},z_{2},R)$$

Thus

(22) $$E\left\{{\hat{F}}^{\left(n_{0}+1\right)}(z_{1},z_{2},R)\right\}=\sum_{j=0}^{n_{0}}\beta^{j}(1-\beta )E\left\{{\tilde{F}}^{\left(n_{0}-j\right)}(z_{1},z_{2},R)\right\}$$

(23) $$\begin{array}{l} E\left\{{\hat{F}}^{\left(n_{0}+1\right)}(z_{1},z_{2},R)\right\}=(1-\beta )F(z_{1},z_{2},R)\sum_{j=0}^{n_{0}}\beta^{j}\\ \;=F(z_{1},z_{2},R)(1-\beta )\frac{\left(1-\beta^{\left(n_{0}+1\right)}\right)}{\left(1-\beta \right)}\\ \;=F(z_{1},z_{2},R)(1-\beta^{\left(n_{0}+1\right)}){|}_{n_{0}\to \infty }\\ \;=F(z_{1},z_{2},R) \end{array}$$

From the above analysis, it can be clearly seen that for any value of 0 *< β <* 1, the algorithm will converge to an unbiased estimator of the original image.

## 4. Results and Discussion

To evaluate the performance of the proposed color image restoration algorithm, we consider the following tasks:

1. Restore a HR image from multiple blurred, LR and noisy “**simulated**” TOMBO color images.
2. Restore a HR image from multiple blurred, LR and noisy images and compare the results with the previous method [9, 10].
3. Restore a HR image from multiple blurred, noisy “**real**” TOMBO color images.

### 4.1. Examples of Simulated Images

In this section, we test the performance of our proposed algorithm in restoring a HR image from simulated TOMBO images of “Lena” [29]. The algorithm performance is compared with the pixel rearrange method developed in [2] for TOMBO imagers. Simulation parameters for the generated LR, blurred and noisy images are given in Table 2 for noiseless and noisy cases. The simulated images are generated f(ollowing the pro)cedure described in [1]. In all simulations, the SNER is defined as $\text{SNER}=10\log\left(\frac{\text{Signal Energy}}{\text{Noise Enegry}}\right)$.

**Figure 4.**
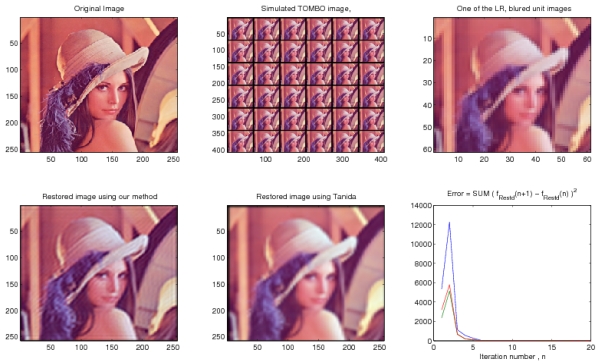
Simulation results, 6 × 6 images, *D =* 4, *L =* 4, *ℓ =* 7, no additive noise.

**Figure 5.**
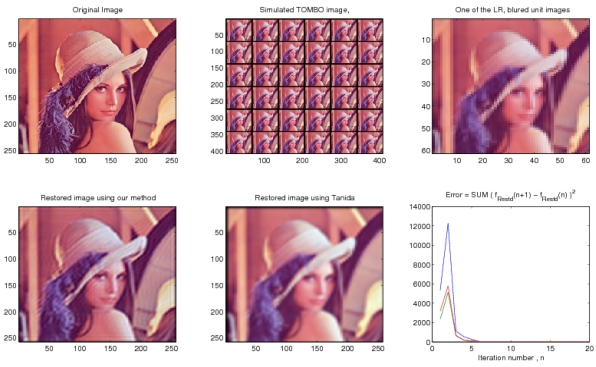
Simulation results, 6 × 6 images, *D =* 4, *L =* 4, ℓ *=* 7, SNER = 4.968 dB.

Figure 4 shows that our restoration algorithm can restore a HR color image from the simulated LR, blurred TOMBO color images in the absence of additive noise. Our method performs better than the pixel rearrange method because the pixel rearrange method can not align captured images. In Figure 5, we tested the algorithm with the simulation parameters given in Table 2, but with additive noise. Our algorithm appears almost insensitive to additive noise, whereas a significant amount of noise can still be seen in the image restored by of the pixel rearrange method. From the two simulation results, we can see that our proposed algorithm converges rapidly.

### 4.2. Comparison with Existing Image Restoration Methods

**Figure 6.**
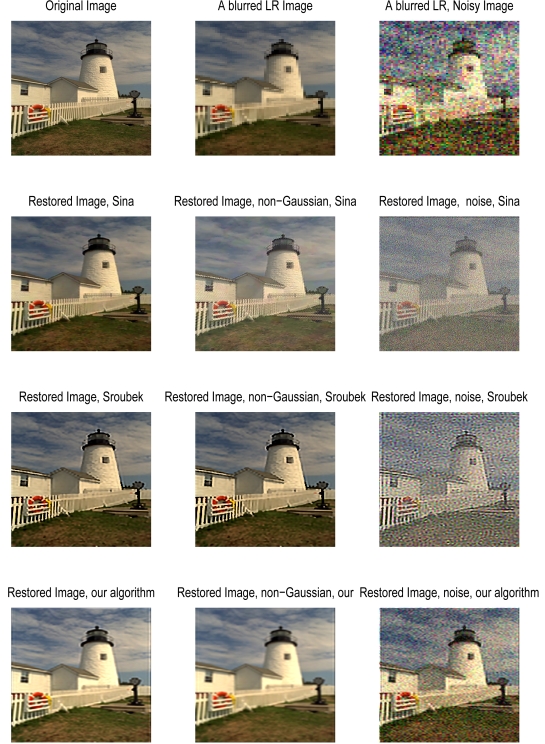
Simulation results for 12 blurred LR images of the lighthouse, in noiseless, and noisy, SNER=10.4616 dB, *D =* 4, *L =* 4, *ℓ =* 5.

In this section, we compare our algorithm with the advanced restoration methods developed by Sina in [9] and Sroubek in [10]. We generated 12 blurred, LR, noiseless and noisy images following the procedure explained in [9]. Results for the two cases are shown in Figure 6, and the PSNR [9] for each restoration is summarized in Table 3.

At high SNRs, our algorithm does not perform as well as Sina's, which enforces more constraints including (i) a penalty term to enforce similarities between the raw data and the HR estimate (data fidelity penalty term), (ii) a penalty term to encourage sharp edges in the luminance component of the HR image (spatial luminance penalty term), (iii) a penalty term to encourage smoothness in the chrominance component of the HR image (spatial chrominance penalty term), and (iv) A penalty term to encourage homogeneity of the edge location and orientation in different color bands (inter-color dependencies penalty term). In Sroubek's method, the following constraints are enforced: (i) a fidelity term, (ii) a smoothing term using variational integrals, (iii) a consistency term that binds the different volatile PSFs, (iv) a smoothing term to overcome the higher nullity of integer-factors, and (v) an anisotropic term for edge preservation. Although our algorithm only imposes two fundamental constraints, its performance is visually satisfactory, as seen in Figure 6. Our method achieved a PSNR of 16.348 dB in contrast to a PSNR of 21.986 dB and 18.250 dB for Sina's and Sroubek's methods, respectively. A higher PSNR, however, does not always correlate to subjective quality [30, 31]. For instance, the Shift-And-Add restored image (in Sina *et al.* [9] page 148) has a PSNR of 17.17 dB, but is clearly of poor quality compared to the one we restored. At low SNRs (10.46 dB), our algorithm visually outperforms both aforementioned methods (Figure 6). However, their PSNRs (14.13 dB for Sina's and 13.78 dB for Sroubek's) are higher than the 10.89 dB achieved by our algorithm. This can be explained by the fact that both Sina's and Sroubek's methods minimize regularized energy functions.

Furthermore, we have observed that Sina's method experience some limitations when applied to images blurred using semi-gaussian PSFs. This could be due to the gaussian kernel used in Sina's approach. It is also important to point out that Sina's method uses multiple frames of an image with different displacements (i.e. diversity is guaranteed, see section VI in [9]), while our method uses multiple simultaneous observations of the same scene captured by the TOMBO imager. The diversity between all captured images cannot be guaranteed for the case of our TOMBO imager.

### 4.3. Examples of Real Images

In this section, we investigate the performance of our proposed algorithm with real captured TOMBO color images. In this example, the captured images are that of a “teddy bear” picture, with a plan object located at 200 mm from the sensor module. Each unit imager has 60 × 60 pixels and each pixel is 6.25 *μm ×* 6.25 *μm.* The microlens array has the following characteristics: 1.3 mm focal length, 0.5 mm diameter of aperture, and a 0.5 mm pitch for the microlens array. The TOMBO imager integrates a color filter array with a Bayer pattern. Demosaicing is achieved on-chip. To test the performance of the restoration algorithms at lower SNRs, a zero mean white Gaussian noise is added manually to the captured LR images. System parameters for this real example are given in Table 4.

**Figure 7.**
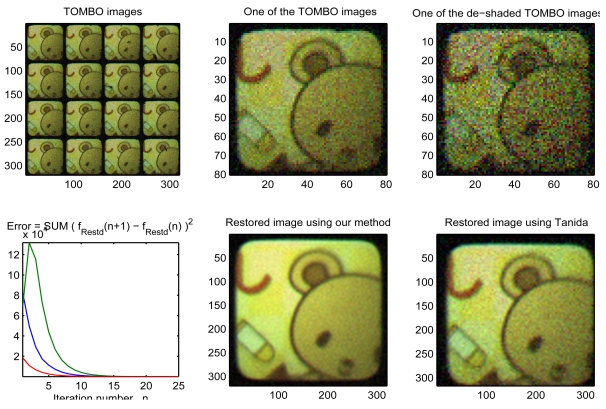
Experimental results, 4 × 4 unit images, *D =* 4, *L =* 4, *ℓ =* 5, SNER = 15.774 dB.

Figure 7 demonstrates that our proposed algorithm is able to restore a HR color image of the “teddy bear” picture using real captured TOMBO LR observations. In addition, our algorithm can significantly minimize the additive noise. It outperforms the conventional pixel rearrange method [33, 34]. Furthermore, our algorithm can restore the HR color image within only 15 iterations.

## 5. Conclusions

A blind color image restoration method is proposed for the reconstruction of HR color images using multiple LR, degraded and noisy color images captured by TOMBO imagers. The proposed spectral-based method only imposes two fundamental image restoration constraints (positivity and support region) to (i) correct the blur that affects the captured LR images, (ii) minimize the interchannel cross-correlations between RGB color components, (iii) significantly reduce the impact of additive noise, and (iv) reconstruct a HR color image. The computation complexity of the algorithm is low compared with existing techniques because it uses FFT for spectral estimation.

The proposed restoration algorithm has a rapid convergence rate of 10 to 20 iterations. Results show that the proposed algorithm is capable of restoring a HR image from the degraded LR color TOMBO images even when the SNER is as low as 5 dB. The proposed algorithm uses FFT and only two fundamental image restoration constraints, which makes it suitable for silicon integration with the TOMBO imager.

## Figures and Tables

**Table 1.** Proposed color image restoration algorithm.

**Step 1**: Set the values of *L, ℓ, α*_1_, *α_2_,β, F_M_, F_∞_*
**Step 2**: Select the color to be restored *ϑ* ε {*R, G, B*}
**Step 3**: Iteration *n =* 1, initialize *f̂* (*x, y, ϑ*), hence *F̂*(*f*_1_, *f*_2_,*ϑ*) = FFT {(*x, y, ϑ*)}
**Step4**:For *i,j* = 1,2, *…, μ* estimate
(24) $${\hat{C}}_{G_{i,j}}F*(f_{1},f_{2},\vartheta )=G_{i,j}(f_{1},f_{2},\vartheta )*\hat{F}*(f_{1},f_{2},\vartheta )$$ (25) $${\tilde{H}}_{i,j}(f_{1},f_{2},\vartheta )=\frac{{\hat{C}}_{G_{i,j}F*}(f_{1},f_{2},\vartheta )-\alpha_{1}}{\hat{F}(f_{1},f_{2},\vartheta )\hat{F}*(f_{1},f_{2},\vartheta )+\alpha_{2}}\Rightarrow {\tilde{h}}_{i,j}(x,y,\vartheta )=\text{IFFT}\left\{{\tilde{H}}_{i,j}(f_{1},f_{2},\vartheta )\right\}$$
**Step 5**: Impose PSF constraints to get the accurate estimates
(26) $${\hat{h}}_{i,j}(x,y,\vartheta )=\{\underset{0}{\left|{\tilde{h}}_{i,j}(x,y,\vartheta )\right|,}\underset{\text{otherwise}}{<x,y>\in \left[\ell ,\ell \right]}\Rightarrow {\hat{H}}_{i,j}(f_{1},f_{2},\vartheta )=\text{FFT}\left\{{\hat{h}}_{i,j}(x,y,\vartheta )\right\}$$
**Step 6**: Estimate the biased image spectra
(27) $$\tilde{F}(f_{1},f_{2},\vartheta )=\frac{\sum_{i=1}^{\mu }\sum_{j=1}^{\mu }{\hat{C}}_{G_{i,j}H_{i,j}^{*}}(f_{1},f_{2},\vartheta )-\alpha_{1}}{\sum_{i=1}^{\mu }\sum_{j=1}^{\mu }{\hat{C}}_{H_{i,j}H_{i,j}^{*}}(f_{1},f_{2},\vartheta )+\alpha_{2}}\Rightarrow \tilde{f}(x,y,\vartheta )=\text{IFFT}\left\{\tilde{F}(f_{1},f_{2},\vartheta )\right\}$$
**Step 7**: Impose the image constraints
(28) $$\overset{⌣}{f}(x,y,\vartheta )=\{\begin{array}{cc} \left|\tilde{f}(x,y,\vartheta )\right| & <x,y>\in \left[LM,LN\right]\\ F_{M}, & \left|\tilde{f}(x,y,\vartheta )\right|\geq F_{\infty },<x,y>\in \left[LM,LN\right]\\ 0 & \text{otherwise} \end{array}$$
then estimate the original image by updating the estimates using
(29) $$\hat{f}(x,y,\vartheta )=\beta \hat{f}(x,y,\vartheta )+(1-\beta )\overset{⌣}{f}(x,y,\vartheta )$$
**Step 8**: Scale the estimated images pixels or use histogram normalization to find *a* and *b*, then adjust the image using
(30) $$\hat{f}(x,y,\vartheta )=a\times \frac{\left(\hat{f}(x,y,\vartheta )-{\hat{f}}_{\text{min},\vartheta }\right)}{\left({\hat{f}}_{\text{max},\vartheta }-{\hat{f}}_{\text{min},\vartheta }\right)}+b$$
**Step 9**: Repeat from **Step 4** until convergence, then repeat for another color *ϑ*

**Table 2.** Input parameters for simulated TOMBO images.

	*μ* × *μ*	*M × N*	SNER	***ℓ***	*LM ×LN*	**α_1_**	α_2_	***β***	# Iterations
Figure 4	6 × 6	60 × 60	-	7	240 × 240	0.1	10	0.1	20
Figure 5	6 × 6	60 × 60	4.968 dB	7	240 × 240	0.1	10	0.1	20

**Table 3.** PSNR values (in dB) for the restoration methods.

	Sina [9]	Sroubek [10]	This Work
Noiseless	21.986	18.250	16.348
Noisy	14.13	13.78	10.89

**Table 4.** Input parameters for real captured TOMBO images, *D =* 4.

	μ × μ	*M × N*	SNER	*ℓ*	*↑L*	*LM*	*× LN*	α_1_1	α_2_	*β*	# of Iterations
Figure 7	4 ×4	60 × 0	15.774 dB	5	4	240	40	0.001	0.001	0.9	25
